# MicroRNA-26a negatively regulates toll-like receptor 3 expression of rat macrophages and ameliorates pristane induced arthritis in rats

**DOI:** 10.1186/ar4435

**Published:** 2014-01-14

**Authors:** Congshan Jiang, Wenhua Zhu, Jing Xu, Bo Wang, Weikun Hou, Rui Zhang, Nannan Zhong, Qilan Ning, Yan Han, Hongchuan Yu, Jian Sun, Liesu Meng, Shemin Lu

**Affiliations:** 1 Department of Biochemistry and Molecular Biology, School of Basic Medical Sciences, Xi’an Jiaotong University Health Science Center, West Yanta Road No.76, Xi’an, Shaanxi 710061, PR China; 2 Key Laboratory of Environment and Genes Related to Diseases, (Xi’an Jiaotong University), Ministry of Education, Xi’an, Shaanxi, PR China; 3 Department of Bone and Joint Diseases, Hong Hui Hospital, Xi’an Jiaotong University Health Science Center, Xi’an, Shaanxi 710054, PR China; 4 Department of Respiratory Medicine, Xi’an Children Hospital, Xi’an, Shaanxi 710003, China; 5 Department of Epidemiology and Health Statistics, School of Basic Medical Sciences, Xi’an Jiaotong University Health Science Center, Xi’an, Shaanxi, China

## Abstract

**Introduction:**

Abnormal toll-like receptor (TLR)3 signaling plays an indispensable role in pathogenesis of both experimental and human rheumatoid arthritis, and microRNAs (miRNAs) might orchestrate this signaling pathway. This study was performed to determine the relationship between miR-26a and *TLR3* in rat macrophages and to observe effects of miR-26a mimic on pristane induced arthritis (PIA) in rats.

**Methods:**

Dual luciferase reporter assay was used to validate the direct interaction between miR-26a (a candidate miRNA to target *tlr3* mRNA) and *tlr3* 3′UTR. MiR-26a regulation on *TLR3* gene expression was determined using RT-qPCR and Western blotting after miR-26a mimics and inhibitors were transfected into rat macrophage line NR8383 cells. Poly I:C (*TLR3* ligand) was used to trigger *TLR3* activation, and mRNA expression of its downstream cytokines interferon (*ifn*)-β and tumor necrosis factor (*tnf*)-α was accordingly detected to determine the regulation of TLR3 signaling. Expressions of *TLR3* and miR-26a were detected during rat bone marrow derived macrophage (BMDM) induction, in pristane stimulated NR8383 cells and spleens from methotrexate (MTX) treated PIA rats. A miR-26a mimic was administrated intraperitoneally to PIA rats, and arthritis severity was evaluated by macroscopic or microscopic observations.

**Results:**

Direct target relationship between miR-26a and *tlr3* mRNA in rats was confirmed. Modifications of miR-26a function by transfection of miR-26a mimics and inhibitors exhibited corresponding repression and augmentation of *TLR3* and its signaling downstream cytokine expressions in NR8383 cells. The alteration of miR-26a expression was negatively related with *TLR3* expression during BMDM induction, in pristane-primed NR8383 cells and PIA rat spleens. Moreover, both abnormal expressions were rescued in MTX treated arthritis rat spleens. The miR-26a mimic treatment displayed the depression of *TLR3* expression and ameliorated the disease severity in the rats with pristane induced arthritis.

**Conclusions:**

MiR-26a negatively regulates *TLR3* signaling via targeting of *TLR3* itself in rat macrophages, and this finding provides a novel insight into abnormal *TLR3* overexpression during experimental arthritis.

## Introduction

Toll-like receptors (TLRs) belong to a member of the pattern-recognition receptor family that recognizes highly conserved structural motifs from microbial pathogens known as pathogen-associated molecular patterns, or from necrotic and dying cells known as damage-associated molecular patterns. Stimulation of TLRs by binding with corresponding ligands triggers at least two distinct signaling pathways: an MyD88-dependent pathway and an MyD88-independent pathway. TLRs are expressed mainly in innate immunocytes and play a crucial role in defending microbial invaders. Recently, accumulating data have documented that TLRs are also an important player in the development of inflammatory and immune diseases such as rheumatoid arthritis (RA), asthma, diabetes and atherosclerosis [1,2]. Among TLRs, TLR3 recognizes double-stranded RNA as its ligand, activates IFN regulatory factor 3 (IRF3) and IRF7 through a specific MyD88-independent signaling cascade and triggers the expression of target cytokine genes including IFN-β and TNF-α [3-5]. Recent studies have demonstrated that TLR3 is involved in the pathogenesis of virus infection and autoimmune disorders, especially RA, in which RA synovial fibroblasts (RASFs) from early-stage patients highly express TLR3 and react with its ligand *in vitro*, suggesting that this pathway is activated early in the disease process [6,7]. RASFs are activated by stimulation with both synthetic and endogenous TLR3 ligands such as poly I:C and necrotic RA synovial fluid cells, resulting in pro-inflammatory gene expression [8]. The activated TLR3 pathway could further promote RASFs sustaining B cell activation in the synovium [9]. In the previous study, we found that both TLR3 mRNA and protein expressions are prominently upregulated in splenic macrophages in rats with pristine-induced arthritis (PIA) and collagen-induced arthritis (CIA), and downregulation of *TLR3* expression modulates the severity of arthritis [10,11]. *TLR3* in the synovium of PIA rats is also overexpressed in an early and persistent style and the activation of the *TLR3* signaling pathway *in vivo* could aggravate PIA [12]. The findings indicate that excess and persistent expression of the TLR3 gene in macrophages and synovial cells could be responsible for arthritis development.

TLR3, like other TLRs, has long been considered remarkably conserved across the taxonomic kingdoms and constitutively expressed by numerous immune cells [13], even though studies on regulation of the TLR3 signaling pathway have been widely performed [11,14-16]. Our study and others have shown that TLR3 expression per se changes dramatically under certain scenarios and regulation to its expression is a means to prevent the excess production of proinflammatory cytokines from its overactivated signaling pathway. We presume that miRNA as an important regulator participates in orchestrating the gene expression-relevant TLR3 and its signal molecules.

MiRNAs are defined as endogenous approximately 22 nt RNAs that play a crucial regulatory role via binding to the mRNAs of protein-coding genes to mediate post-transcriptional repression [17]. Recent studies have mainly focused on the miRNA roles in TLR signaling molecules rather than their role in modulating the expression TLR3 itself [18]. For example, miR-223 regulates TLR-triggered IL-6 and IL-1β production by targeting Signal transducer and activator of transcription (STAT3) [19] and miR-146 exerts negative feedback regulation of TLRs and cytokine receptor signaling via targeting IL-1 receptor-associated kinase (IRAK)1 and TNF receptor-associated factor (TRAF)6 [20]. Aforementioned research into miRNA is necessarily profound, and indicates the possibility of miRNA participating in arthritis via regulation of TLR signaling. However, the direct target interaction between miRNA and TLR3 has been underestimated, and miRNA regulation of TLR3 and its signaling during arthritis development remains an enigma. The present study was performed to find the potential miRNAs that can target the TLR3 molecule itself, verifying both the miRNA and TLR3 expression in macrophages during differentiation and pristane stimulation, as well as in the spleen of PIA rats, and observing the effects of an miR-26a mimic on *TLR3* expression and arthritis severity in PIA rats.

## Methods

### Bioinformatics

The rat *tlr3* mRNA sequence was obtained from GenBank [NM_198791]. TargetScan 6.2 [21] and MiRanda [22,23], two widely advocated bioinformatic software systems, were chosen to seek the candidate miRNAs according to the presence of binding sites in the seed region, efficacy of targeting and probability of conserved targeting. The unanimous predictive outcome from two algorithms was used for further investigation.

### Dual luciferase assay

A 198-bp-long *tlr3* 3′UTR element containing the putativebinding site of miR-26a (miR-26a sequence: 5′-UUCAAGUAAUCCAGGAUAGGCU-3′) was cloned downstream of the luciferase gene between the SacI and HindIII sites within the pMIR-Report™ Luciferase (Ambion, Austin, USA) vector to construct a pMIR-TLR3 vector. The mutated *tlr3* 3′UTR element containing site mutations at numbers 2, 4, and 6 in the putative miR-26a:*tlr3* seed-pair region was obtained using the PCR-directed mutation method and cloned into the same vector, namely the mutated pMIR-TLR3 vector. The pRL-TK vector (Promega, Fitchburg, USA) served as a control. Plasmids were prepared with the EZNA™ Endo-free Plasmid Maxi Kit (Omega Bio-tek, Norcross, USA). The constructs were sequenced to prove sequence integrity (Genscript company, Nanjing, China).

Hela cells cultured in DMEM high glucose medium (Hyclone, Logan, USA) containing 10% FBS (Hyclone) were used for dual luciferase reporter assay. Briefly, both the firefly pMIR-Report™ Luciferase (Ambion) and renilla pRL-TK (Promega) vectors (90 ng:10 ng per well) were transfected into Hela cells (2 × 10^4^ cells per well seeded for 24 h before transfection) simultaneously with 10 nM miR-26a mimic/inhibitor (GenePharma, Shanghai, China) or negative control (NC) using Lipofectamin 2000™ (Invitrogen, Carlsbad, USA) transfection reagents in a 48-well culture plate. Sequences from 5′ to 3′ end are listed as follows: NC mimics sense UUCUCCGAACGUGUCACGUTT, anti-sense ACGUGACACGUUCGGAGAATT; miR-26a mimics sense UUCAAGUAAUCCAGGAUAGGCU, anti-sense CCUAUCCUGGAUUACUUGAAUU; NC inhibitor CAGUACUUUUGUG UAGUACAA (2′Ome-modified), miR-26a inhibitor AGCCUAUCCUGGAUUACUU GAA (2′Ome-modified).

The lucifease activity was detected using Dual-Luciferase® Reporter 1000 Assay System (Promega) by a plate-reading luminometer (Luminoskan ascent 392, Thermo, Waltham, USA) 24 h after transfection, and the relative luciferase activity value was achieved against the renilla luciferase control.

### Bone marrow-derived macrophage (BMDM) induction

Rat primary bone marrow-derived cells were isolated from three normal DA rats, and seeded at the density of 2 × 10^6^/ml in L929-conditioned medium to differentiate into macrophages as described in Cold Spring Harbor Protocols [24]. Attached cells on days 0, 3 and 6 were harvested for miR-26a and *TLR3* expression analyses.

### Pristane stimulation in macrophages

NR8383 cells, a rat macrophage cell-line, were cultured in F-12 K medium (Sigma-Aldrich, St. Louis, USA) containing 15% FBS (Hyclone). MiRNA mimics or inhibitors were transfected using Lipofectamin™ 2000 (Invitrogen). Emulsion of pristane (ACROS Organics, New Jersey, USA) was made by repeated aspiration with medium. For single pristane stimulation, 5 × 10^5^ cells per well were seeded for 24 h before a 50-μl pristane emulsion was added in the culture medium (final concentration 1 mM), and harvested after stimulation for 24 h. Furthermore, NR8383 cells were incubated with the mimic or inhibitor for 24 h prior to activation of *TLR3* signaling by stimulation of pristane or poly I:C (TLR3 ligand, 10 μg/ml, Amersham Biosciences, Amersham, UK) for another 24 h, and then harvested for analysis. Appropriate mimic and inhibitor dose for transfection was decided by pilots (data not shown), in which 10 nM was found to be sufficient, hence, was chosen for most of the following procedures.

### Pristane-induced arthritis in rats

DA rats were housed under specific pathogen-free conditiona. Eight rats at the age of 8 to 12 weeks were randomly divided and used in each group. Arthritis was induced by a single intradermal injection with 150 μl pristane at the base of the rat’s tail [25]. In methotrexate (MTX) treated PIA rats, 0.25 mg intraperitoneal (i.p.) MTX/kg per rat was administered in 200 μl saline on days 8, 10 and 12, and rats were sacrificed on day 20 after pristane induction [26]. The same volume of saline was injected into PIA rats to serve as the saline-treated PIA group. The rats without pristane injection or MTX treatment served as the control group. Arthritis development and severity was monitored every two to four days by the perimeters of the foot pad, and macroscopic scoring until sacrifice. After sacrifice the spleens were collected and stored at −80°C for RNA quantification.

### MiR-26a mimic treatment in pristane induced arthritis rats

MiR-26a miR-Up™ agomir molecule, which was cholesterol-modified at the 3′ end, with two phosphorthioations at the 5′ end and four at the 3′ end, and methylation for all skeletons, was purchased from the company (GenePharma, China) and used as a miR-26a mimic. The NC agomir molecule and solvent saline were used as controls. All three groups each contained seven age- and sex-matched DA rats. Arthritis was induced in rats using pristane at day 0 and then rats were treated with miR-26a mimic, NC mimics or saline (150 μg/kg, equal to 11.4 nmol/kg molecules dissolved in saline each time) through i.p. injection four times, on days 8, 12, 15 and 19. Arthritis severity was scored every other day using a comprehensive scoring system [25] until sacrifice, and the perimeters of ankle, foot pad and body weights were measured every four days. Rats were sacrificed on day 23 after pristane injection, and ankles were collected and prepared for H&E staining. Pathological changes included synovitis, joint destruction and repair and were scored from 0 to 3 for each of the three parts [10]. Spleens were harvested and stored at −80°C for RNA and protein detection. Rat plasma was separated for determination of TNF-α using the ELISA method and nitric oxide (NO) detection using the Griess method [27]. The animal experiment was approved by the Institutional Animal Ethics Committee, and procedures also conformed to the Institutional Animal Care and Use Committee (IACUC) of Xi’an Jiaotong University.

### RT-qPCR

A total RNA of 500 ng isolated with Trizol® Reagent (Invitrogen) was used in an miRNA-specific stem loop reverse transcription (RT) reaction for miRNAs, and 5 μg for the RT reaction using oligo d(T) primer. cDNA was synthesized by RevertAid™ First Strand cDNA Synthesis Kit (Fermentas). Real-time quantitative PCR (qPCR) was performed by iQ5 system (Bio-rad) with SYBR® Premix Ex Taq™ II (TaKaRa) for quantification. Triplicates were used for the test in each sample. Gene and miRNA expression was normalized by glyceraldehyde-3-phosphate dehydrogenase (GAPDH) and U6 snRNA, respectively. Purity of PCR products was confirmed using a melting curve, and all data were analyzed using the 2^-ΔΔCt^ (relative quantification) method. The information about genes, primer sequences (synthesized by Genscript company), and annealing temperatures is depicted in Table 1.

**Table 1:** **Genes and primers for RT-qPCR and 3**′ **UTR cloning**

**Gene symbol (accession number)**	**Primer name**	**Annealing temperature (°C)**	**Sequences (from 5′ to 3′)**
*TLR3* [GenBank:NM_198791]	Sense	60	GATTGGCAAGTTATTCGTC
	Antisense		GCGGAGGCTGTTGTAGG
	3′UTR sense	60	CGAGCTCTTTGGAGTCAGTGAAGGGAT
	3′UTR antisense		GAAGCTTCCCATGTATTTATTTGGAGCAA
	3′UTR mutation sense	58	GTCTGAGTTTTTCATCAAAGTTTGTAT
	3′UTR mutation Antisense		ATACAAACTTTGATGAAAAACTCAGAC
*GAPDH* [GenBank:NM_017008]	Sense	65	CGGCAAGTTCAACGGCACAG
	Antisense		GAAGACGCCAGTAGACTCCACGAC
*IFNB* [GenBank:NM_019127]	Sense	54	CTTGGGTGACATCCACGACTAC
	Antisense		GGCATAGCTGTTGTACTTCTTGTCTT
*TNFA* [GenBank:NM_012675]	Sense	60	TCAGCCTCTTCTCATTCCTGC
	Antisense		TTGGTGGTTTGCTACGACGTG
*rno-miR-26a* [miRBase: MIMA0000796]	RT	-	GTCGTATCCAGTGCAGGGTCCGAGGTATTCGCACTGGATACGACAGCCTA
	Sense	60	CGCTTCAAGTAATCCAGGA
	Antisense		GTGCAGGGTCCGAGGT
*U6 snRNA* [GenBank:K00784]	Sense	60	CTCGCTTCGGCAGCACA
	Antisense		AACGCTTCACGAATTTGCGT

### Western blotting

Total cell lysates were prepared and subjected to SDS/PAGE gel according to standard procedures in the Bio-rad system. GAPDH on the same membrane was used as a loading control. Rabbit anti-*TLR3* antibody (Biosen, Beijing, China) and mouse anti-GAPDH antibody (Abcam) were used as the primary antibody, and the signal was further detected using the secondary antibody of goat anti-rabbit or goat anti-mouse immunoglobulin (Ig)G labeled with horseradish peroxidase (HRP). Signal intensity was determined by Supersignal® West Pico kit (Thermo Scientific).

### TNF-α determination

Cell supernatant and rat plasma were collected, and *TNF*-α was determined using the ELISA development kit (Peprotech, USA). Briefly, 100 μl plasma or supernatant was added onto the TNF-α antibody-coated plate and incubated at 25°C for 2 h. After adding the biotin-conjugated detecting *TNF*-α antibody and incubating for 2 h, streptavidin-HRP was added and 3,3'-5,5' tetramethylbenzidin (TMB) was used for development. The optical density (OD) value was obtained at the wave of 450 nm by multiskan spectrum (Thermo, USA). The complete medium of F12K + 15% FBS was used as a blank, and the *TNF*-α concentration was calculated from the standard curve, which was obtained using the series dilution of recombinant rat *TNF*-α from 3,000 pg/ml to zero.

### Statistics

Quantitative data were expressed as mean ± standard error of the mean (SEM), and statistical analysis of differences between experimental groups was performed by the Mann–Whitney *U*-test. Differences with *P*-values less than 0.05 were considered as statistically significant.

## Results

### Putative targeting relationship between miR-26a and TLR3 in rats was confirmed by dual luciferase reporter gene assay

**Figure 1:**
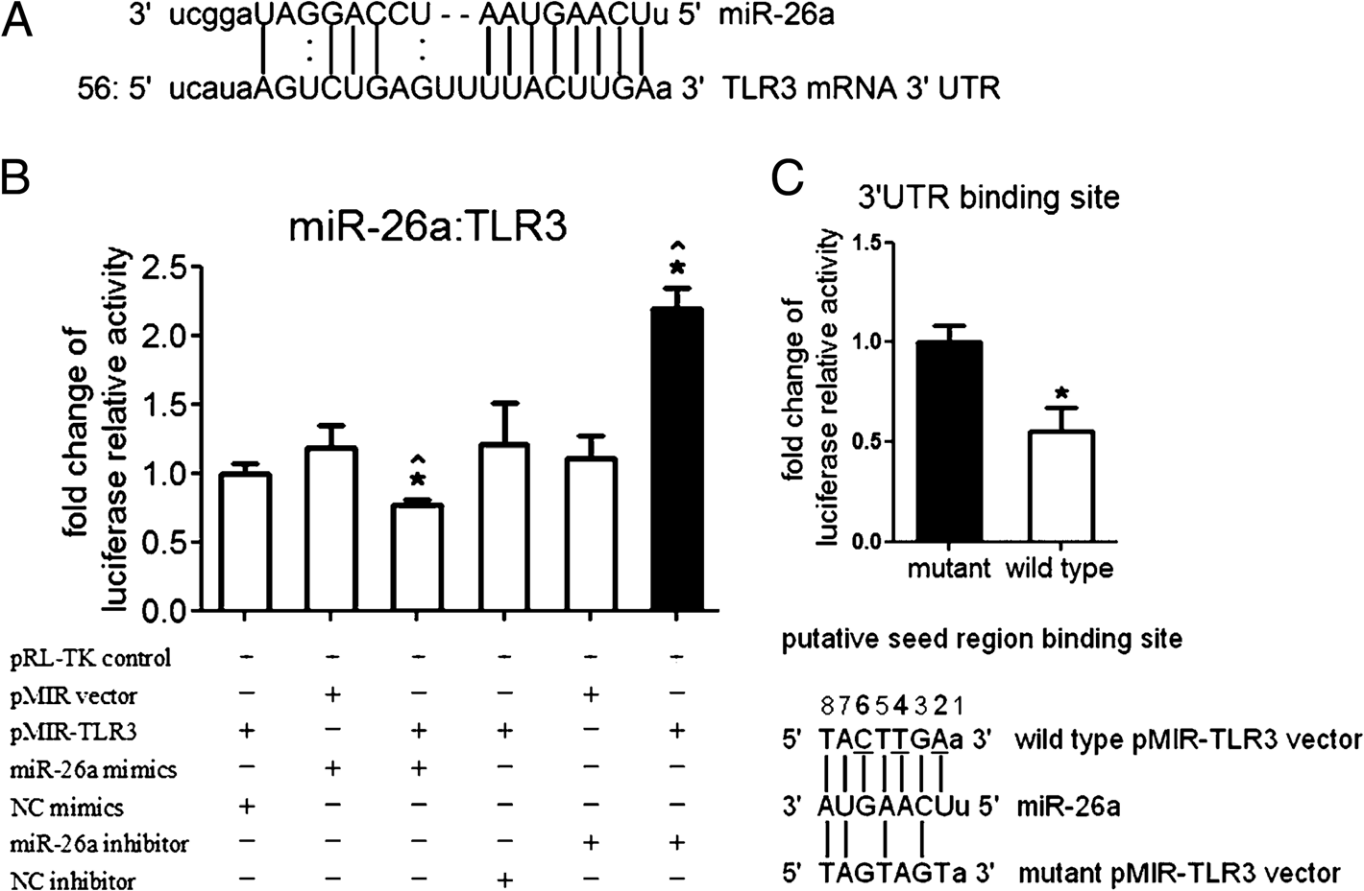
**Putative target relationship between miR-26a and toll-lke receptor (TLR)3 in rats. (A)** Schematic diagram of pairing relationship between miR-26a and *tlr3* mRNA 3′UTR in rats. The rat *tlr3* mRNA 3′UTR sequence contains a putative binding site of miR-26a analyzed by the bioinformatics software [28]. **(B)** Effect of miR-26a mimics/inhibitor on the luciferase activity of pMIR-TLR3 vector. Target relationship between *tlr3* mRNA 3′UTR and miR-26a was analyzed by dual luciferase reporter assay, pMIR-REPORT™ Luciferase vectors with or without *tlr3* mRNA 3′UTR element (pMIR-TLR3 or pMIR empty vectors) were transfected into Hela cells, and pRL-TK vector was used as an internal control reporter in all conditions for normalization. NC, negative control; pMIR, pMIR-REPORT™ Luciferase vector. Bars represent standard error of the mean (SEM) from three transfections. Experiments were independently repeated twice. *Statistically significant difference (*P* <0.05) compared with NC miRNA; ^significant difference compared with vector (Mann–Whitney *U*-test). **(C)** Effect of miR-26a mimics on the luciferase activity of wild-type or mutated pMIR-TLR3 vector. pMIR-REPORT™ Luciferase vector carrying a TLR3 mRNA 3′UTR element with the putative binding site of miR-26a was transfected into Hela cells (upper panel). Bars represent SEM from three experiments. *Statistically significant difference (*P* <0.05, Mann–Whitney *U*-test). Schematic diagram of pairing relationship between miR-26a and wild-type or mutated pMIR-TLR3 vector indicates that three nucleotides have been altered in the mutated pMIR-TLR3 vector (lower panel).

Bioinformatics results showed that miR-26a and miR-340-5p were candidate miRNAs for targeting rat *TLR3* (Figure 1A). As it could bind to *tlr3* mRNA from diverse species, including bushbabies, mice, rabbits and armadillos, miR-26a was chosen for further investigation.

To confirm whether *TLR3* is the target of miR-26a, the firefly and renilla dual luciferase reporter assay was performed in Hela cells (Figure 1B). Transfecting both miR-26a mimics and pMIR-TLR3 vector into Hela cells could lead to a significant reduction (*P* <0.05) of luciferase activity by 20% on average compared with the NC mimics or by 35% compared with the empty pMIR vector. On the contrary, the miR-26a inhibitor significantly elevated (*P* <0.05) the luciferase activity of pMIR-TLR3 vector by 70% on average compared with the NC inhibitor or by 80% compared with the empty pMIR vector. To further verify this specific binding, a mutated pMIR-TLR3 vector with a three-nucleotide mutation in the putative seed-binding site was constructed and transfected together with miR-26a mimics and pRL-TK into Hela cells (Figure 1C). Compared with the mutated pMIR-TLR3 vector, there was a significant reduction (*P* <0.05) of luciferase activity after the wild-type pMIR-TLR3 vector and miR-26a mimics were transfected into cells together with the pRL-TK control, suggesting that miR-26a specifically binds to the 3′ UTR of rat TLR3 mRNA.

### MiR-26a could negatively regulate TLR3 signaling by intervening in miR-26a function in macrophages

**Figure 2:**
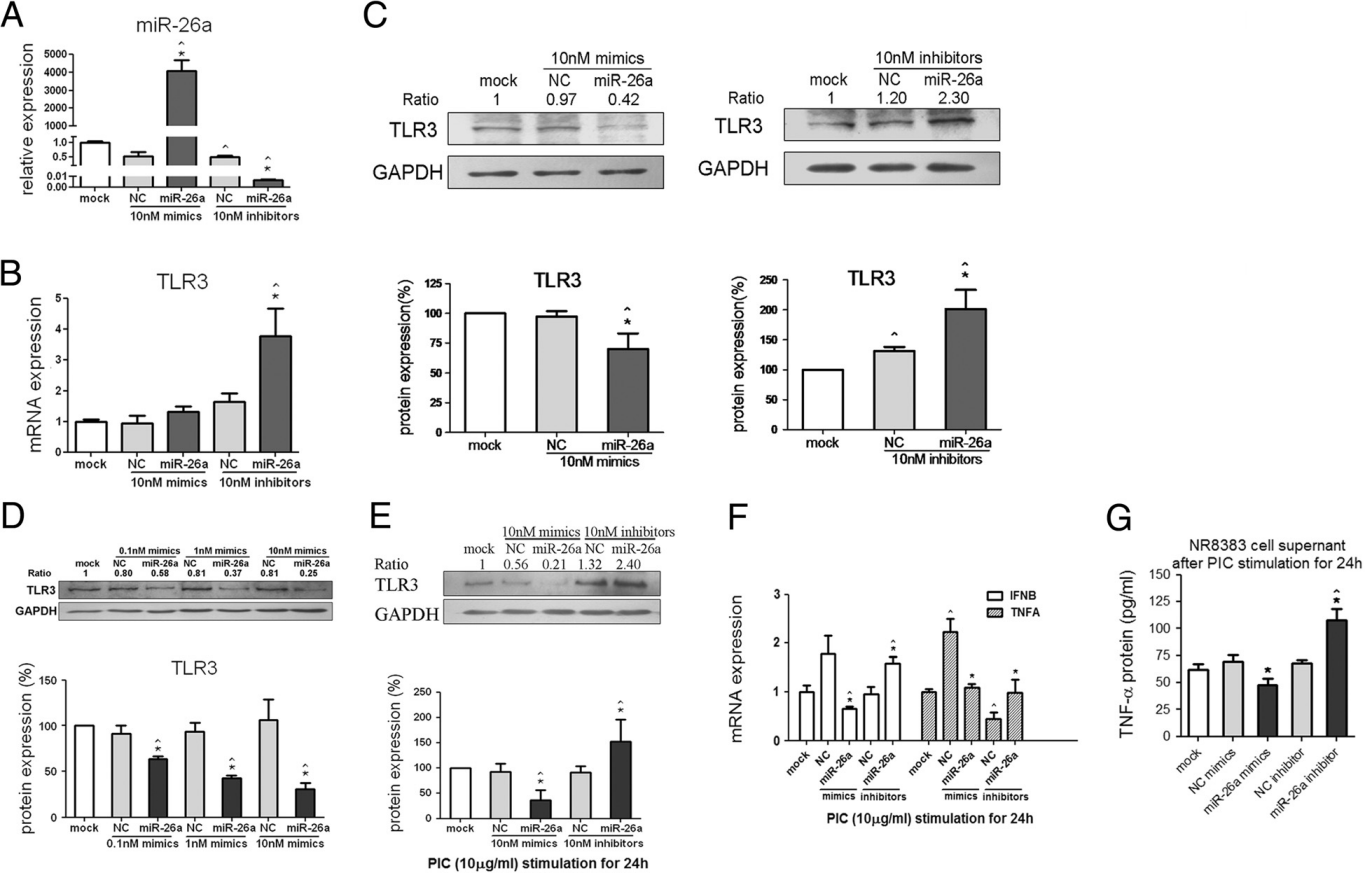
**Effects of miR-26a on toll-like receptor (TLR)3 signaling by gain or loss of miR-26a function in NR8383. (A)** Stem loop RT-qPCR results of miR-26a expression, **(B)** RT-qPCR results of *tlr3* mRNA expression, and **(C)** western blotting of *TLR3* protein expression after 10nM miR-26a mimics or inhibitor was transfected into NR8383 macrophages for 48 h. **(D)** Western blotting results of *TLR3* protein expression after 0.1, 1.0 and 10.0 nM of miR-26a mimics were transfected into NR8383 cells for 48 h. **(E)** Western blotting of *TLR3* protein expression and **(F)** RT-qPCR results of *ifn*-β and *tnf*-α mRNA expression in NR8383 and ELISA results of *TNF*-α protein expression in cell supernatant **(G)** after incubated with 10 nM mimics and inhibitors for 24 h and poly I:C (PIC, TLR3 ligand, 10 μg/ml) stimulation for another 24 h. U6 snRNA and glyceraldehyde-3-phosphate dehydrogenase (*GAPDH*) were used as internal controls in RT-qPCR for miRNA and mRNA expression detection respectively. Bars represent the standard error of the mean from three cell experiments. NC, negative control. One representative plot and quantitative data from three independent western blotting experiments are shown. Ratio indicates the optical intensity of TLR3 protein bands against *GAPDH*. *Statistically significant differences (*P* <0.05) against the NC; ^significant differences against the mock group (Mann–Whitney *U*-test).

NR8383 cells, a macrophage cell line, were transfected with miR-26a mimics and miR-26a was significantly increased (as much as 4,000 times) respectively, in the miR-26a mimics group compared with the NC (*P* <0.05) or mock group (*P* <0.05). The cells were transfected with miR-26a inhibitors and miR-26a expression was suppressed by 99% compared with the NC (*P* <0.05) or mock (*P* <0.05) group, suggesting that a gain or loss of miR-26a function occurred (Figure 2A). TLR3 mRNA expression results showed that miR-26a mimics hardly affected *tlr3* mRNA expression, however miR-26a inhibitors were able to raise *tlr3* mRNA expression level by 3.7- or 1.9-fold respectively compared with the mock (*P* <0.05) or the NC (*P* <0.05) group (Figure 2B). In the mean time, western blotting results of *TLR3* protein expression showed that 10nM miR-26a mimics were able to significantly suppress *TLR3* protein expression by approximately 30% on average compared with the mock (*P* <0.05) or the NC group (*P* <0.05), and 10nM miR-26a inhibitors sharply increased *TLR3* protein expression by 100% compared with the mock (*P* <0.05) or by 70% compared with the NC (*P* <0.05) (Figure 2C). Different doses of miR-26a mimics were transfected into NR8383 cells to confirm the translational suppression. Responding to this increasing miR-26a expression, *TLR3* protein expression displayed dose-dependent inhibition by approximately 30%, 50% and 70% respectively, compared with the NC group (Figure 2D).

To find out whether miR-26a could control *TLR3* signaling, NR8383 were incubated with 10 nM mimics or inhibitors for 24 h prior to activation of *TLR3* signaling by poly I:C stimulation for another 24 h, and then harvested for expression analysis. After the signaling pathway was turned on by its ligand, the protein expression of *TLR3* and mRNA expression of *ifn*-β and *tnf*-α, two specific downstream cytokines, were detected. The results showed that miR-26a mimics caused a 60% reduction, whereas inhibitors caused a 1.5-fold increase of *TLR3* protein on average compared with both the NC and mock group (Figure 2E). MiR-26a mimics caused a 60% and 30% reduction of *ifn*-β mRNA compared with the NC (*P* <0.05) or mock (*P* <0.05), and a 100% reduction of *tnf*-α mRNA compared with the NC (*P* <0.05). miR-26a inhibitors caused a 60% increase of *ifn*-β mRNA compared with both (*P* <0.05) the NC and mock, and a 100% increase of *tnf*-α mRNA compared with the NC (*P* <0.05) (Figure 2F). ELISA results also showed that the *TNF*-α protein concentration in the cell supernatant was also significantly suppressed after miR-26a mimic treatment compared with the NC (*P* <0.05), and enhanced after inhibitor treatment compared with both the mock and NC groups (both *P* <0.05) (Figure 2G).

### MiR-26a was downregulated and TLR3 was upregulated during the induction of rat BMDM

**Figure 3:**
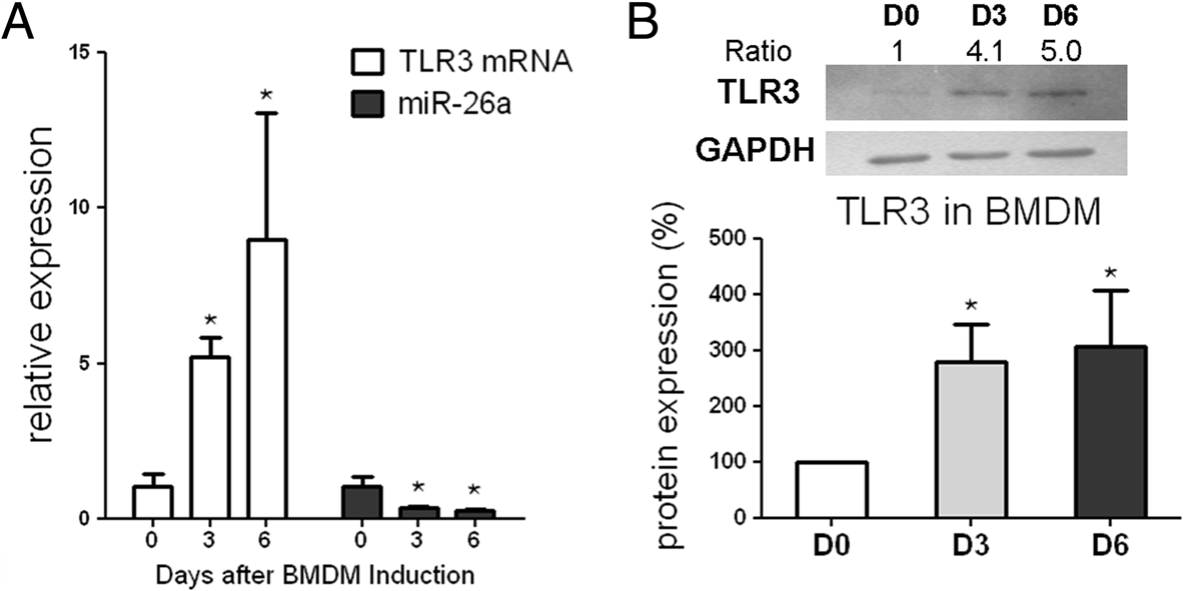
**miR-26a and toll-like receptor (TLR)3 expression during bone marrow-derived macrophage (BMDM) induction. (A)** RT-qPCR results of *tlr3* mRNA and miR-26a expression and **(B)** western blotting results of *TLR3* protein expression on day (D)0, D3 and D6 during BMDM induction. Bone marrows were obtained from three DA rats. U6 snRNA and glyceraldehyde-3-phosphate dehydrogenase (*gapdh*) were used as internal controls in RT-qPCR for miRNA and mRNA expression detection, respectively. Bars represent the standard error of the mean from three rats. *Statistically significant differences (Mann–Whitney *U*-test, *P* <0.05)*.* One representative plot and quantitative data from three independent western blotting tests are shown. Ratio indicates the optical intensity of *TLR3* protein bands against GAPDH.

MiR-26a and *TLR3* expression was monitored after rat BMDM was induced for 0, 3 and 6 days. Along with macrophage induction, *tlr3* mRNA was upregulated 5- and 9-fold, whereas the miR-26a expression declined by 60% and 70% respectively on days 3 and 6 compared with day 0 after BMDM induction (Figure 3A). TLR3 protein expression also increased 2.8- and 3.0-fold on average during BMDM induction (Figure 3B).

### MiR-26a and TLR3 expression displayed an opposite trend in rat macrophages after pristane stimulation

**Figure 4:**
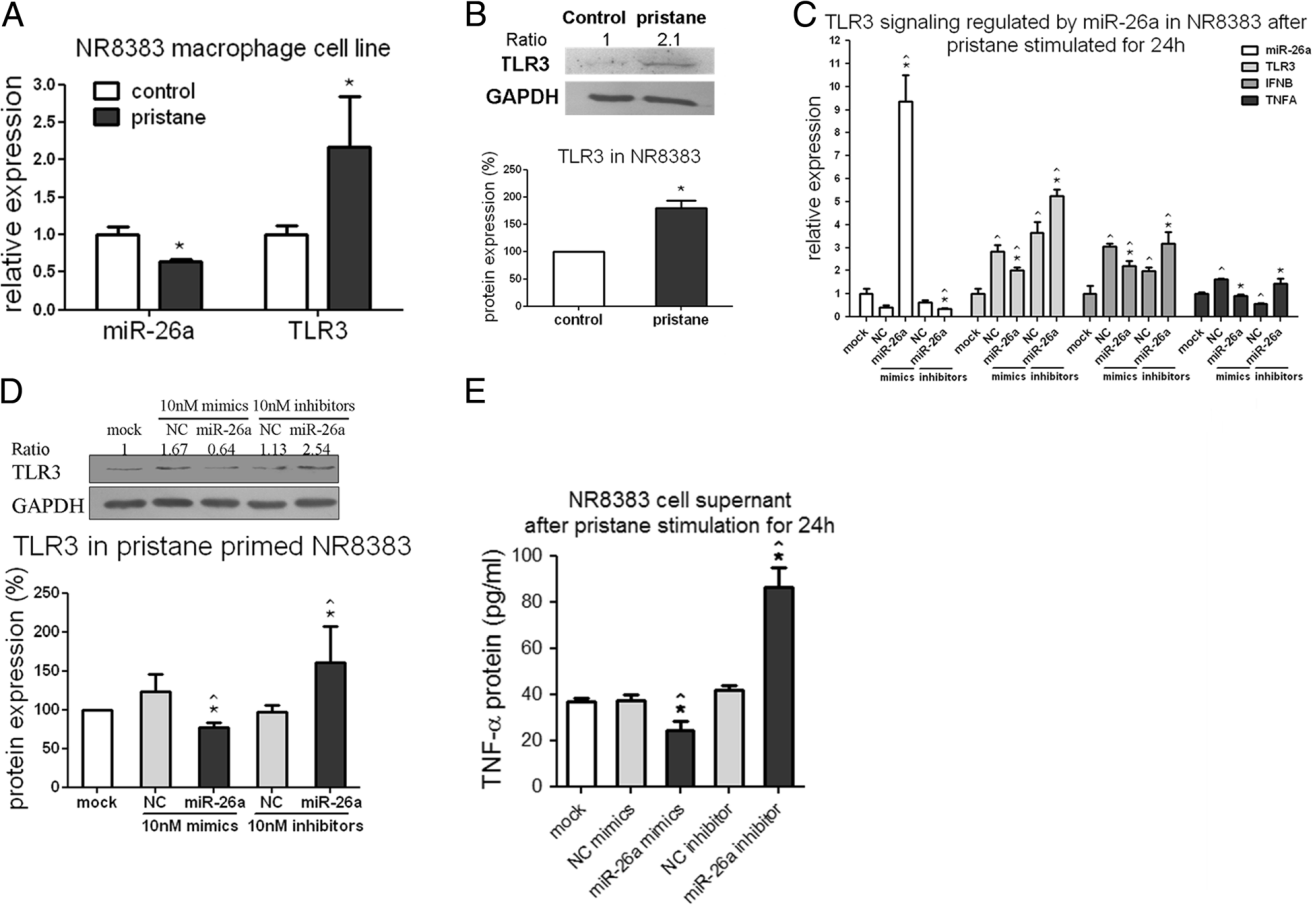
**miR-26a and toll-like receptor (TLR)3 expression in rat macrophages after pristane stimulation *in vitro*. (A)** RT-qPCR results of *tlr3* mRNA and miR-26a expression and **(B)** western blotting results of *TLR3* protein expression in NR8383 after stimulated by 1 mM pristane emulsion for 24 h. **(C)** RT-qPCR results of miR-26a, *tlr3*, *ifn*-β and *tnf*-α mRNA expression, **(D)** western blotting results of *TLR3* protein expression in NR8383 and **(E)** ELISA results of *TNF*-α protein expression in the cell supernatant after incubation with 10 nM mimics and inhibitors for 24 h and 1 mM pristane stimulation for another 24 h. U6 snRNA and glyceraldehyde-3-phosphate dehydrogenase (*GAPDH*) were used as internal controls in RT-qPCR for miRNA and mRNA expression detection, respectively. Bars represent the standard error of the mean from three experiments. *Statistically significant differences compared with medium control in **(A)** and **(B)**. In **(C)** and **(D)**, *significant differences compared with the negative control miRNA group; ^significant differences compared with the mock group (Mann–Whitney *U*-test, *P* <0.05)*.* One representative plot and quantitative data from three independent western blotting tests are shown. Ratio indicates the optical intensity of *TLR3* protein bands against *GAPDH*.

In pristane-primed NR8383 cells, enhanced expression of *tlr3* mRNA (*P* <0.05) and protein expression approximately 2-fold compared with the medium control, whereas miR-26a expression decreased by 40% on average (*P* <0.05) after 24 h pristane stimulation (Figure 4A and B). The incubation with miR-26a mimics/inhibitors was performed for a further 24 h and pristane stimulation for another 24 h to confirm the target repression of TLR3 signaling by miR-26a in macrophages. Successful transfection was confirmed by miR-26a expression monitored by RT-qPCR, and the results showed that alteration of miR-26a function could regulate *TLR3* signaling after pristane stimulation in macrophages. miR-26a mimics and inhibitors, respectively, caused a 30% reduction in 40% increase of *tlr3* mRNA (*P* <0.05), a 30% reduction or 60% increase in *ifn*-β mRNA (*P* <0.05), and a 45% reduction or 2.5-fold increase in *tnf*-α mRNA (*P* <0.05) compared with the NC group (Figure 4C). Both double-stranded mimics and single-stranded inhibitors of miR-26a or the NC could activate *tlr3* and *ifn*-β mRNA compared with the mock (*P* <0.05). The NC mimics increased, whereas the inhibitors decreased *tnf*-α mRNA expression (*P* <0.05). MiR-26a mimics exhibited corresponding repression of *TLR3* protein by 40% and 25% compared with the NC or mock group, whereas miR-26a inhibitors increased *TLR3* expression 1.6-fold compared with the NC or mock (Figure 4D). Similarly, *TNF*-α protein concentration in the cell supernatant was detected using ELISA, and the results showed that it was significantly suppressed (*P* <0.05) after miR-26a mimic treatment, and enhanced (*P* <0.05) after inhibitor treatment compared with the mock or NC group (Figure 4E).

### Implication of miR-26a found in PIA rat spleens

**Figure 5:**
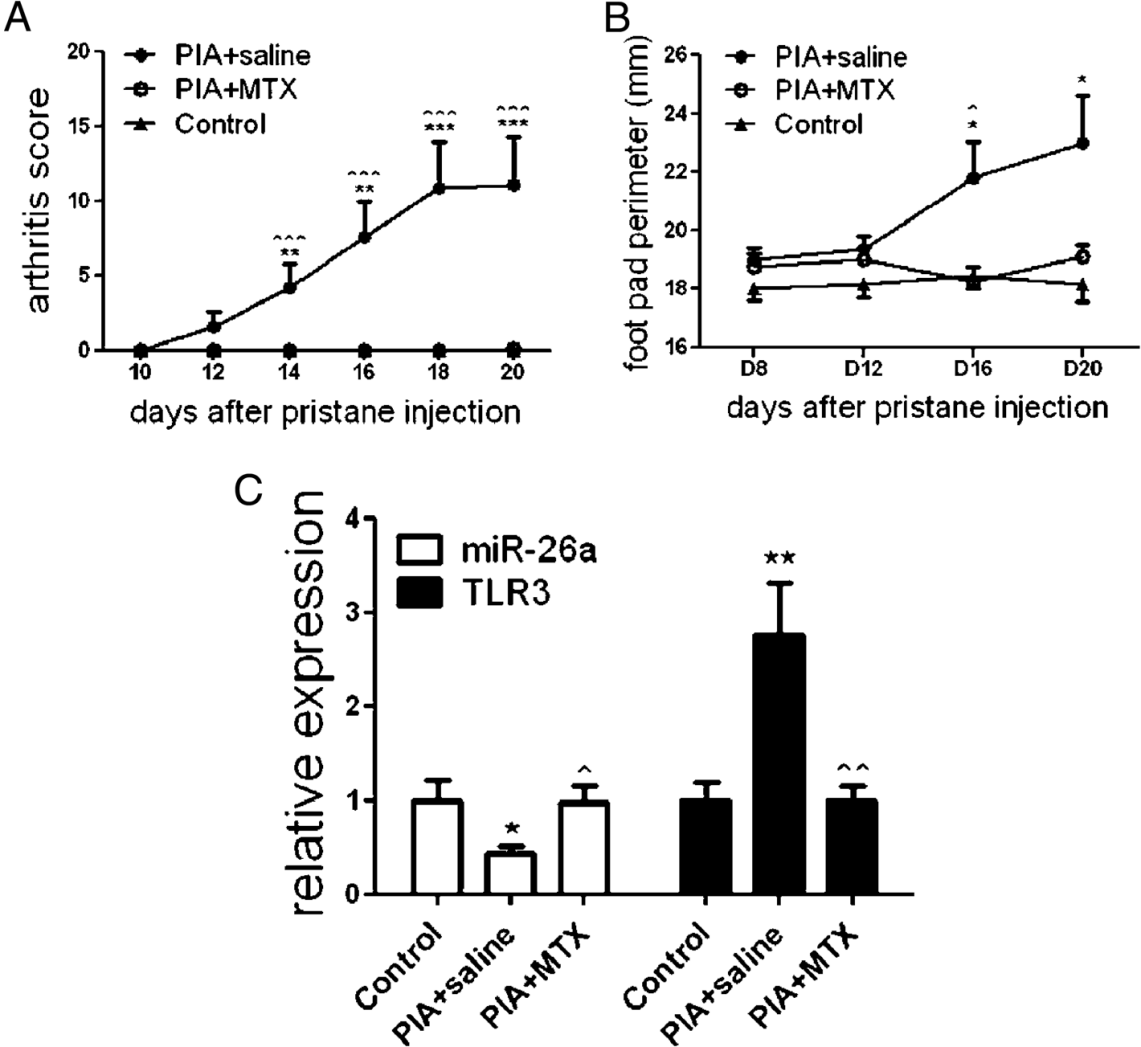
**miR-26a and toll-like receptor (TLR)3 expression in pristine-induced arthritis (PIA) rats with and without treatment with methotrexate (MTX). (A)** Arthritis scores and **(B)**, foot-pad perimeter in PIA rats with and without treatment with MTX. **(C)** RT-qPCR results of *tlr3* mRNA and miR-26a expression in rat spleens from control, saline-treated PIA and MTX-treated PIA group on day 20 after pristane injection. U6 snRNA and glyceraldehyde-3-phosphate dehydrogenase (GAPDH) were used as internal controls in RT-qPCR for miRNA and mRNA expression detection, respectively. Bars represent the standard error of the mean from eight rats used in each group. *Statistically significant differences, (Mann–Whitney U-test), *PIA plus saline against control; ^PIA plus saline against PIA plus MTX; *,^*P* <0.05; **,^^*P* <0.01; ***,^^^*P* <0.001.

The arthritis score (Figure 5A) and foot-pad perimeter (Figure 5B) in saline-treated PIA rats were significantly different from control or MTX-treated PIA rats, and there was no statistical difference between MTX-treated PIA and control rats, suggesting that MTX could abrogate arthritis. Expression of *tlr3* and miR-26a was monitored in PIA rat spleens and the results showed that *tlr3* mRNA expression was sharply upregulated 3-fold (*P* <0.01), whereas miR-26a expression significantly decreased by 60% on average. However, both *tlr3* excess expression and miR-26a reduction after MTX treatment surprisingly recovered to the levels of control rats (Figure 5C).

### MiR-26a mimic can ameliorate pristine-induced arthritis in rats

**Figure 6:**
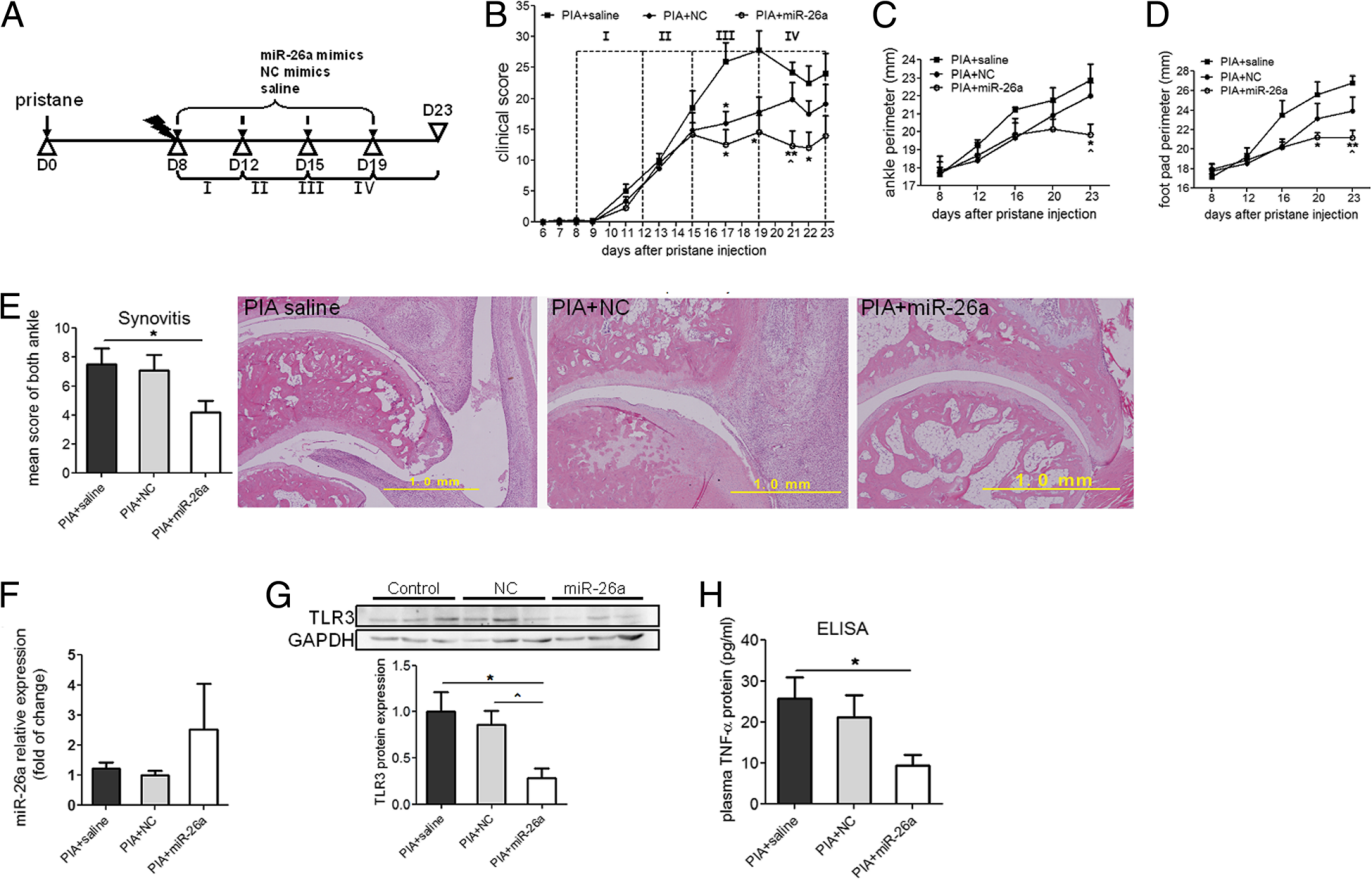
**Evaluation of miR-26a mimic treatment in pristine-induced arthritis (PIA) rats. (A)** A brief scheme for miR-26a mimic treatment in PIA rats. D, day. **(B)** Arthritis clinical score curve from the onset day to D23. **(C)** Mean ankle perimeter and **(D)** mean foot-pad perimeter of rats. **(E)** Quantative data for pathological scores indicating synovitis of arthritis (left panel) and representative H&E staining of rat ankle from each group (right panel), scale:1.0 mm. **(F)** miR-26a expression detected by stem loop qRT-PCR in spleens of miR-26a-treated rat spleens. U6 snRNA was chosen as THE endogenous control. **(G) ***TLR3* protein expression detected by western blotting. Upper bands show representative plots of toll-like receptor (*TLR*)3 and endogenous glyceraldehyde-3-phosphate dehydrogenase (*GAPDH*) control using protein samples from rat spleen. Lower figure shows the quantitative data for the protein expression ratio calculated using rat spleen protein samples from all groups. **(H) ***TNF*-α protein expression detected by ELISA in rat plasma from all groups. Error bar represents mean ± standard error of the mean of each group (n = 8). *Statistically significant differences compared with PIA + saline control; ^significant differences compared with PIA + negative control (NC): *,^*P* <0.05; **P <0.01 (Mann–Whitney *U*-test).

To observe whether miR-26a overexpression *in vivo* can influenze arthritis severity, PIA rats were treated with miR-26a mimic, NC mimics and saline four times until rats were sacrificed (Figure 6A). The arthritis clinical score showed that miR-26a could not prevent the occurrence of arthritis from the beginning, but could significantly restrain the arthritis severity after the third injection on day 15 till the rats were sacrificed on day 23 (Figure 6B). Ankle (Figure 6C) and food-pad perimeter (Figure 6D) in the PIA + miR-26a group was significantly lower than in the PIA + saline or PIA + NC group on day 23, indicating relief of joint-swelling after miR-26a mimic treatment (Additional file 1). Body weight loss after arthritis was also alleviated (Additional file 1). There was no significant difference in the organ-/body-weight ratio in the spleen, inguinal lymph nodes, heart, liver, lung or kidney, indicating therapy in both the NC and miR-26a miRNA (Additional file 1). Three major pathological indexes of arthritis in rat ankles, such as synovitis, joint destruction and joint repair were evaluated, and the results showed that miR-26a mimics can reduce synovitis in the PIA + miR-26a group compared with the PIA + saline group (Figure 6E). There was no significant difference in the total pathological change or joint destruction and joint repair (Additional file 1). Meanwhile, rat spleens were harvested for RNA and protein expression. MiR-26a expression in spleens from the PIA + miR-26a group remained 2.5 times higher than in the NC group, even after the last mimic administration four days previously (Figure 6F). *TLR3* protein expression in the spleen was significantly suppressed in the PIA + miR-26a group compared with the PIA + NC group or PIA + saline group (Figure 6G). The ELISA test also showed that the plasma TNF-α in PIA + miR-26a rats was lower than in the PIA + saline rats (Figure 6H). However, there was no significant difference in NO in rat plasma among the groups (Additional file 1). These results indicated that miR-26a mimic finely controlled *TLR3* protein expression and ameliorated arthritis severity in the PIA rats.

## Discussion

To sum up, we predicted miR-26a to be a candidate to target *TLR3* in rats and many other mammals. This putative targeting relationship between miR-26a and *TLR3* was further confirmed by dual reporter gene assay. In addition, miR-26a was verified to be involved in the negative regulation of *TLR3* signaling by targeting *TLR3* itself in macrophages, and modifications of miR-26a function exhibited corresponding repression or augmentation of *TLR3* signaling. In BMDM induction and pristane-stimulated NR8383 cells, miR-26a reduction was found to be responsible for *TLR3* overexpression in rat macrophages. MiR-26a expression was downregulated as *tlr3* expression was decreased in spleens of PIA rats, and both were rescued after MTX treatment in arthritic rats. MiR-26a mimic was administrated to PIA rats, and the results showed that *TLR3* protein expression was suppressed, and the arthritis severity alleviated. Our finding not only discloses the deregulation of miR-26a in *TLR3* expression, but also offers a novel and reliable mechanism for abnormal *TLR3* overexpression in experimental arthritis.

According to miRBase [29], an authoritative miRNA database, miR-26a belongs to one of the miRNA families broadly conserved with perfectly identical sequences among vertebrates. In previous reports, miR-26a was on the list of the top 10% of miRNAs constitutively expressed at a high level in rat spleen [30], and also found to be considerable abundant in rat articular cartilage using Solexa sequencing from our previous study [31]. Its outstanding sufficiency in major immune organs and cartilage suggests its potential implication in arthritis development. Previous studies on miR-26a have provided much evidence of this miRNA as an important regulator in cell proliferation and differentiation. For example, it has been reported that miR-26a plays a crucial role in regulating mouse hepatocyte proliferation during liver regeneration [32], and it could also modulate osteogenic differentiation of human adipose tissue-derived stem cells by targeting SMAD1 transcription factor [33]. In addition, upregulated miR-26a promotes myogenesis by post transcriptional repression of Ezh2, a known suppressor of skeletal muscle cell differentiation [34].

Mir-26a genes are present on chromosome 3p22.2 and 12q14.1 in the human genome and 8q32 in the rat genome, and mir-26a itself could be regulated. Microarray-based miRNA expression profiling found that *MYC* oncogene could repress miR-26a [35], Trastuzumab could induce mir-26a and hence, produces therapeutic actions in human epidermal growth factor receptor-2 (HER2)-positive breast cancer cells [36], C/EBP-α can directly activate mir-26a expression during mechanical stretch, which leads to hypertrophy of human airway smooth-muscle cells [37], and menin, a transcriptional factor has been demonstrated by chromatin immunoprecipitation (ChIP) to occupy the mir-26a gene promoter, thus inducing its expression, and confirming its role as a positive regulator of mir-26a [38].

In bioinformatics, we found that miR-26a targets *TLR3* in the rat, mouse, rabbit, bushbaby and armadillo; however, the binding pattern of *TLR3*:miR-26a disappears in the human genome with two nucleotide mutations at the seed region compared with the rat genome. MiR-26a also putatively targets TLR4 in humans, and in the chimpanzee, rhesus monkey, horse, elephant, tree shrew and tenrec. This profile is interestingly complementary among vertebrates available in the database. Moreover, both TLR3 and TLR4 with an unique ability to activate IRF-3 and promote the expression of type I IFN and downstream proinflammatory cytokines [39], are considered the most overwhelming players in RA development [6,40,41]. It seems that miR-26a regulation may transition from TLR3 to TLR4 in many other species. Peer scientists have long held an opinion that various regulation systems including miRNAs might not be able to work in the regulation of TLR expression as in that of most other genes. However, according to our work, both the regulation of *TLR3* pathway mediators and *TLR3* itself by miRNAs should play a crucial role in *TLR3* signaling, which leads to timely and appropriate control of the proinflammatory events.

*TLR3* is intrinsically expressed in rodent macrophages, hence, in this work we chose the rat macrophage cell line NR8383 to explore the expression regulation of the TLR3 gene after miR-26a mimics or inhibitors were transfected into the cells. The negative regulation of the *TLR3* gene from miR-26a was revealed in inactive NR8383 macrophages, further in primary macrophages during BMDM induction, and also in pristine-stimulated NR8383 macrophages, confirming that miR-26a could control TLR3 signaling in rat macrophages. In the inactivated phase, miR-26a mimics hardly affected *tlr3* mRNA, yet repressed its protein by 30%, whereas miR-26a inhibitors increased *tlr3* mRNA 1.9-fold, and protein by 70% on average compared with the NC. Inhibitor treatment was found to cause a much more potent influence on *TLR3* than the mimics. More interestingly, after *TLR3* signaling activation, this negative regulation from miR-26a seemed to be amplified. After pristane activation, miR-26a mimics repressed *tlr3* mRNA by 30% and protein by 40%, and its inhibitors also increased *tlr3* mRNA by 40% and protein 1.6-fold. There is an explanation for these findings, namely that miRNAs act as buffers against variation in gene expression. In this case, endogenous miR-26a might be sufficient for buffering *TLR3* expression fluctuation in inactivated macrophage so that miR-26a inhibitor treatment exhibits a more powerful function than its mimics. This evidence supports the leading opinion of more important roles for miRNAs in conferring robustness to ongoing biological processes [42]. Rescued miR-26a reduction and *TLR3* overexpression in spleens from MTX-treated PIA rats compared with saline-treated ones also suggested the implication of miR-26a in rat arthritis. At the end of this study, the miR-26a administration in PIA rats demonstrated that miR-26a overexpression can suppress *TLR3* protein expression *in vivo*. Such intervening can also lead to the alleviation of arthritic conditions, such as joint swelling and synovitis, which suggests the therapeutic potential of miRNA in TLR overexpression-induced pathological inflammation.

## Conclusion

We found reduction of miR-26a expression in rat macrophages during BMDM induction, pristane stimulation and in spleens of PIA rats in which *TLR3* was overexpressed. MiR-26a-mimic administration also could lead to suppression of *TLR3* protein expression and ameliorate arthritis in PIA rats. These findings demonstrate that miR-26a regulates the *TLR3* signaling pathway by targeting *TLR3* expression, and implicates miR-26a as a drug target for inflammatory suppression in arthritis therapy.

## Abbreviations

BMDM: bone marrow-derived macrophage; bp: base pairs; CIA: collagen-induced arthritis; DMEM: Dulbecco's modified Eagle's medium; ELISA: enzyme-linked immunosorbent assay; FBS: fetal bovine serum; GAPDH: glyceraldehyde-3-phosphate dehydrogenase; H&E: hematoxylin and eosin; HRP: horseradish peroxidise; IFN: interferon; Ig: immunoglobulin; IRF: interferon regulatory factor; IL: interleukin; miRNA: microRNA; MTX: methotrexate; NC: negative control; NO: nitric oxide; OD: optical density; PIA: pristane induced arthritis; RA: rheumatoid arthritis; RASF: rheumatoid arthritis synovial fibroblast; RT-qPCR: reverse transcription-quantitative polymerase chain reaction; SEM: standard error of the mean; snRNA: small nuclear RNA; TLR: toll-like receptor; UTR: untranslated region.

## Competing interests

The authors declare that they have no competing interests.

## Authors’ contributions

CJ, WZ, LM and SL conceived and designed the experiments and SL and LM obtained funding for the study. CJ performed the experiments, analyzed the data and accomplished this paper. LM and WZ assisted in the experiments with both theoretical and technical guidance throughout the entire work. JX assisted in cell culture and the animal model. WH, BW and JS participated in the animal model. NZ and RZ selflessly shared their detailed experimental experience and helped carry out miRNA experiments. YH, QN and HY prepared basic reagents and participated in experimental arrangements. SL, LM, and WZ had extensive scientific discussion throughout this study and participated in manuscript writing. All authors read and approved the final manuscript.

## Supplementary Material

Additional file 1 **Figure showing other arthritis-parameter changes after miR-26a mimic treatment in pristine-induced arthritis (PIA) rats. (A)** Representative arthritis pictures in rats. **(B)** Body weight change. **(C)** Organ weight/body weight ratio. **(D)** Total pathological score, score of joint destruction and repair. **(E)** Plasma nitric oxide (NO) concentration. PIA rats were divided into three groups: PIA + saline, PIA + negative control (NC) and PIA + miR-26a. Error bar represents mean ± standard error of the mean of each group (n = 8). *Statistically significant difference compared with PIA + saline control, *P* <0.05 (Mann–Whitney *U*-test). Click here for file
