# Supplementary material for: MicroRNA-26a negatively regulates toll-like receptor 3 expression of rat macrophages and ameliorates pristane induced arthritis in rats
Source: Arthritis Res Ther. 2014 Jan 14;16(1):R9. doi: 10.1186/ar4435 (PMC3978458; doi:10.1186/ar4435)
Supplement: Additional file 1 — Figure showing other arthritis-parameter changes after miR-26a mimic treatment in pristine-induced arthritis (PIA) rats. (A) Representative arthritis pictures in rats. (B) Body weight change. (C) Organ weight/body weight ratio. (D) Total pathological score, score of joint destruction and repair. (E) Plasma nitric oxide (NO) concentration. PIA rats were divided into three groups: PIA + saline, PIA + negative control (NC) and PIA + miR-26a. Error bar represents mean ± standard error of the mean of each group (n = 8). *Statistically significant difference compared with PIA + saline control, P <0.05 (Mann–Whitney U-test). [file ar4435-S1.pdf]

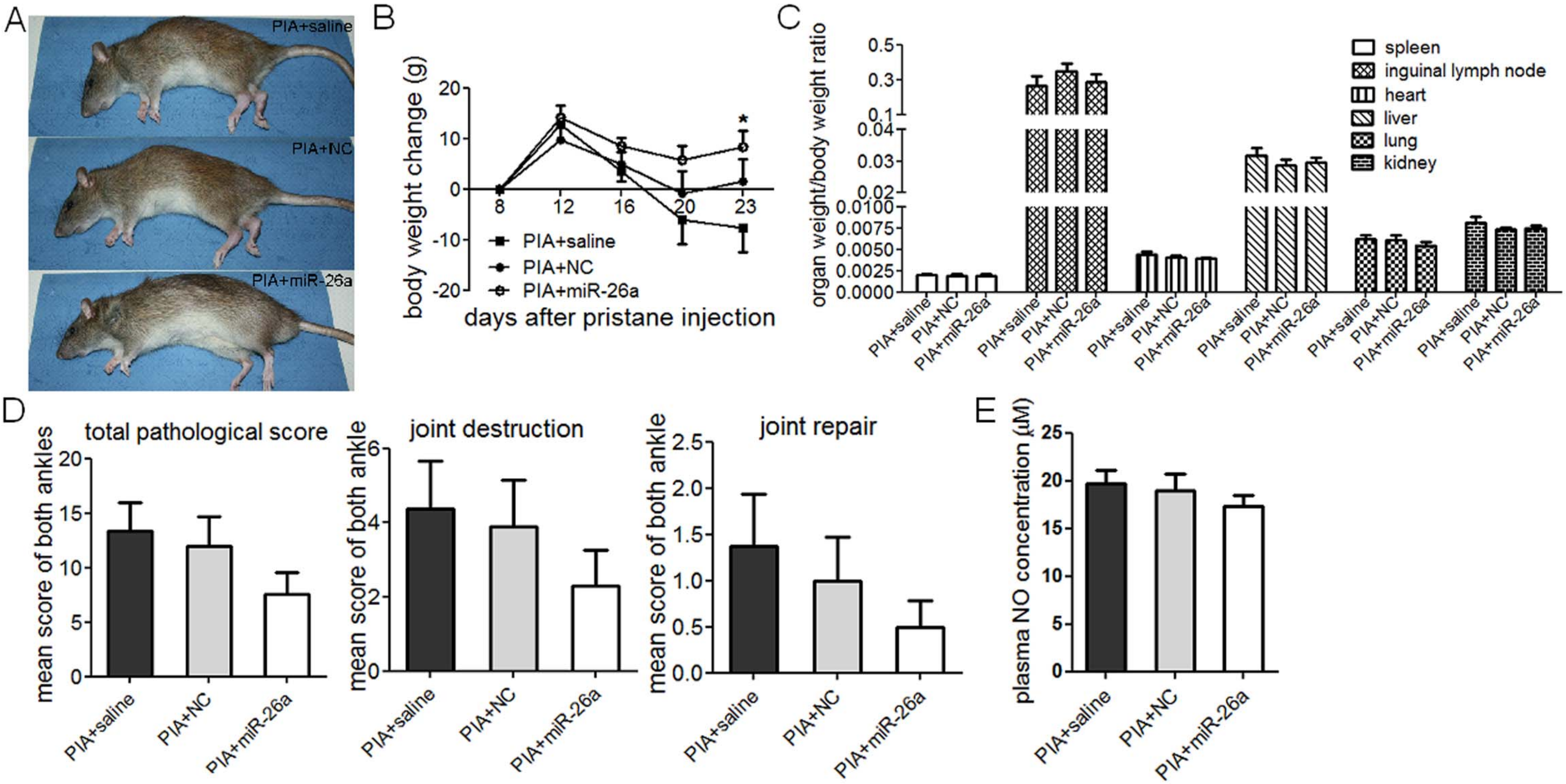

Representative figures (A), body weight change (B), organ weight/body weight ratio (C), total pathological score, score of joint destruction and repair (D), plasma NO concentration (E) in miR-26a treated pristane induced arthritis rat model

Error bar represent mean ± SEM of each group, *: $p<0.05$ compared with PIA+saline control
